# Supplementary material for: Aminobenzoic Acid Derivatives Obstruct Induced Fit in the Catalytic Center of the Ribosome
Source: ACS Cent Sci. 2023 May 30;9(6):1160–9. doi: 10.1021/acscentsci.3c00153 (PMC10311655; doi:10.1021/acscentsci.3c00153)
Supplement: Supplementary file 2 — oc3c00153_si_002.pdf [file oc3c00153_si_002.pdf]

oc-2023-00153e.R1

Name: Peer Review Information for "Aminobenzoic acid derivatives obstruct induced fit in the catalytic center of the ribosome"

First Round of Reviewer Comments

Reviewer: 1

Comments to the Author

In this manuscript, Majumdar et al. use cryo-EM to determine high-resolution structures of ribosomes complexed with charged aminobenzoic acid derivative-charged A-site tRNAs to elucidate how the structural differences in the nonstandard amino acid substrates affect successful docking into the PTC. The three non-L-alpha amino acid monomers studied in this work are incorporated into polypeptides by the wildtype ribosome, but with varying degrees of efficiency, so mapping differences in the structures of ribosomes in complex with these substrates can provide insight into what limits successful incorporation into the PTC. The authors conclude from the structures determined that these monomers prevent the "induced fit" mechanism of U2506 employed for the incorporation of natural amino acids; the aromatic rings of these nonstandard monomers seem to clash with U2506 and prevent its repositioning. This mechanism normally helps to position the nucleophilic α-amine closer to the reactive P-site carbonyl; consequently, the nucleophilic α-amines of these monomers are more distanced from their targets. The nonstandard monomers also appear to disrupt the positioning of water molecules that are thought to be important for the "proton wire" mechanism, likely contributing to a decrease in catalytic activity.

This paper is well written and clearly presents new structures that are of high resolution and will be of interest to the field. While it does not fully explain the trend of why some of the aminobenzoic monomers are better incorporated than others, it provides insight into what the limitations may be and highlights the intricacies of bond formation in the PTC that should be considered when designing for nonstandard amino acid incorporation. I have some minor but essential edits that need to be made.

Minor comments:

1) In Figure 1, the structure for oABZ is cropped differently than for the other two monomers (the squiggly line under "tRNA").

2) In the methods section for Cryo-EM complex preparation, the authors state that they incubated the 70S ribosome complex with 100 µM paromomycin, an antibiotic that interacts with the ribosomal A site (as explored in a previous publication from the Cate lab). I do not understand why paromomycin is included in this incubation, and am not sure if this is a mistake, as it is not discussed anywhere else in the paper or structures and would likely have affected the structures if included. This needs to be clarified.

3) On page 8, it would be helpful in the text to also mention the amine-carbonyl distance for mABZ. This distance is reported in the discussion and shown in the figure but it may be helpful for the reader to be able to include all three values in the text.

4) In the following sentence, "Examples of non-L-α-amino acid monomers that have been introduced into growing polypeptide chains include D-α-amino acids, α-hydroxy and α-thio acids, β2- and β3-amino acids, α-aminoxy and α-hydrazino acids, ...7–15." The authors choose to highlight the work of only one lab (the Suga lab). The authors need to include a broader swath of recent papers and groups in the field, such as: (https://pubs.acs.org/doi/abs/10.1021/jacs.5b12482, https://www.nature.com/articles/s41467-020-18001-x, https://www.nature.com/articles/s41467-022-33701-2,

https://pubs.acs.org/doi/10.1021/acscombsci.0c00179,

https://pubs.acs.org/doi/10.1021/jacs.8b11521,

https://pubs.acs.org/doi/full/10.1021/jacs.5b03135,

https://pubs.acs.org/doi/10.1021/ja035141q)

5) It seems that this paper should be discussed in relation to the current work: https://www.pnas.org/doi/10.1073/pnas.1424712112.

Reviewer: 2

Comments to the Author

The paper describes how aminobenzoic acid derivatives attached to tRNA in the A site of the ribosome alter the conformation of the peptidyl transferase center (PTC), thereby potentially inhibiting peptide bond formation. The resolution of the cryo-EM structures is high, which allows the authors to place the reactive groups as well as the key nucleotides in the PTC, showing that with aminobenzoic acid derivatives the PTC remains in an uninduced conformation that does not facilitate catalysis. Also the positions of waters that presumably take part in the reaction is changed. The paper is clearly written and the conclusions are generally justified. However, several critical issues have to be addressed prior to publication.

1. The authors mention the differences in reactivity between Apy, oABZ and mABZ and take this information into account when discussing their models. However, the respective data are not shown. Specifically, no time course of the reaction is shown for any of the derivatives, which is a significant weakness of the paper. The differences in the rate of catalysis between the L-aa and the non-natural derivatives have to be documented by showing the relative time courses of the reaction.

2. Some of the predictions of the authors' models could be tested by relatively simple biochemical experiments. For example, the suggestion that the disruption of water coordination in the PTC may affect the reaction can be tested using KSIE.

3. For the design of the cryo-EM experiments, it is not entirely clear why there was a need to produce non-hydrolysable tRNA derivatives, as the aminobenzoic acid derivatives are expected to react slowly, which would allow rapid freezing of the cryo-EM sample before any reaction could occur. In Fig. 3A, it is

not clear why Met-tRNA was used in the P site, rather than fMet-tRNA as in all other structures, and how this affects the positioning of the substrates in the PTC. In general, the potential effect of the NH replacement (used to avoid premature peptide bond formation) on the tRNA positioning in the PTC should be discussed.

4. The authors refer to the water arrangement in the PTC as "proton wire", however, this is not the only model in the literature, as the authors must be aware of, as they cite a publication supporting and alternative "proton shuttle" model (ref 31). The original "proton wire" model is inconsistent with some biochemical data, whereas "proton shuttle" appears to be compatible with most of the experimental data. I am not sure whether the actual positions of the water molecules are different in the two models, but the mechanism of the proton transfer is different. While the authors of this paper are not supposed to dwell into the alternative models of peptide bond formation for L-amino acids, it is still important to acknowledge the existence of different models and to take into account the published biochemical results to interpret the structural data.

Reviewer: 3

Comments to the Author

Cate and co-workers aim to resolve structural models of non-L-∝-amino acids incorporation by the ribosome for the first time. They apply cryo-EM to resolve structures of the incorporation at near-atomic resolution to identify the chemical characteristics of these monomers during translation. Three amino benzoic derivatives were used: 3-aminopyridine-4-carboxylic acid (Apy), ortho-aminobenzoic acid (oABZ), and meta-aminobenzoic acid (mABZ). Their results indicate that the Apy incorporates the highest efficiency, followed by oABZ (5-fold lower), and finally mABZ. Unlike the incorporation of L-∝-amino acids, these aminobenzoic monomers sterically hinder RNA residues at the catalytic core inhibiting the favorable induced-fit conformation needed for incorporation. Additionally, cryo-EM structures reveal disruptions or deviations from “proton-wire” that facilitate nucleophilic attack, leaving group stabilization, and the formation of the tetrahedral intermediate needed for peptide synthesis.

The authors do a good job highlighting key aspects of peptide synthesis and how these unnatural monomers disrupt the orientation needed to catalyze the formation of a peptide bond. Their methods provide detail on how they synthesized the components needed for the studies starting from the aminoacylation of the tRNA to the synthesis of the aminobenzoic derivatives. They also include workflows of how they processed their cryo-EM data in the SI. There is potential to do follow-up experiments to alternatively characterize parts of the pathway that cryo-EM alone may not be able to address. Following are comments to address:

Comments:

1.) Since this paper is under consideration at ACS Central Science, which is meant to target the wide readership of ACS, the authors should consider some more general figure. This could include a zoomed-out version of the part of the ribosome they are referring to when they talk about the distances and the angles between functional groups. Introduction: If the rationale is to use catalysts that can do multi-turnover reactions (or maybe a greener and natural alternative) to make these polymers, there could be

an argument there as to why to start with aminobenzoic derivatives. They explain its uses, but not necessarily why these were useful monomers in their study.

2.) p6, L17. In Fig S12E, it looks like the double-headed gray arrow is pointed to the wrong atom in Apy. As per panels F and G, this end of the arrow should be pointed to the carbonyl C of the aromatic ring of the compound, which is to the right not downward. Upon this change, is the distance still 1.6 Å as marked in the manuscript?

3.) Fig 3B. The dashed line appears to be mispositioned. A per panels C and D, it should go up and to the left so that it connects the nucleophilic amine of Apy and the carbonyl C in the P-site. Upon this change, is the distance still 4.9 Å as marked in the manuscript? This value is used again on p.9 of the ms., so the authors need to be careful. In addition, the BD angles should be provided for B-D. Are they in the 90-110 degree range? This is hinted at on p8 lines 35-37 but not quantified nor are the (presumably negative) implications for poor BD angles on reactivity discussed.

4.) p8, L31. Shouldn't this be Figure 1C?

5.) p9, L1-2. Given the lack of strong structural difference between Apy and oABZ, the authors rightly entertained the notion of different electronic properties. I'd like to suggest two additional considerations. (1) Perhaps the endocyclic N in Apy could perform general base catalysis, or if protonated, general acid catalysis and that could promote reactivity. The endocyclic N in the parent pyridine compound is well known to do this. Such activity might help overcome the observed loss of the water wire in the aromatic reagents despite the authors notion that W1 and W2 may not be required for these compounds. The greater dynamics of Apy might allow a docked Apy to do this or alternatively it could do this through buffer catalysis as shown for the twister ribozyme by Messina and Bevilacqua [1]. (2) For Apy and oABZ, there could be an intramolecular hydrogen bond between the exocyclic amine and the carbonyl. Indeed, such compounds often have reduced boiling points because H-bonding is compensated internally (e.g. p-nitrophenol and o-nitrophenol have boiling points of 279 and 216 oC, respectively). This might not explain the difference in reactivity between Apy and oABZ, but it might explain their lower reactivity relative to the natural case. BTW, how much lower they are is not provided but should be.

[1]Messina KJ, Bevilacqua PC. 2018. Cellular small molecules contribute to twister ribozyme catalysis. J Am Chem Soc 140: 10578-10582. doi: 10.1021/jacs.8b06065

6.) p2,L24 and again on p10, the authors discuss how in the natural case the induced fit "protects the reaction center from the side chain of the A-site monomer". If this "protection" is lost, one might expect the reactive amine to react elsewhere in the ribosome. Did the authors look for unprotected reactivity of the aminobenzoic acid derivatives with the ribosome?

Author's Response to Peer Review Comments:

Reviewer: 1

Recommendation: Publish in ACS Central Science after minor revisions noted.

Minor comments:

1. In Figure 1, the structure for oABZ is cropped differently than for the other two monomers (the squiggly line under “tRNA”).

We thank the reviewer for spotting this difference. We replaced Figure 1 with an updated version where this issue is corrected.

2. In the methods section for Cryo-EM complex preparation, the authors state that they incubated the 70S ribosome complex with 100 μM paromomycin, an antibiotic that interacts with the ribosomal A site (as explored in a previous publication from the Cate lab). I do not understand why paromomycin is included in this incubation, and am not sure if this is a mistake, as it is not discussed anywhere else in the paper or structures and would likely have affected the structures if included. This needs to be clarified.

Paromomycin is an aminoglycoside antibiotic that helps stabilize the tRNA in the A-site and favors the classical non-rotated state of the ribosome (Tsai, A. *et al. Cell Reports* **2013**, *3* (2), 497–508.). Since the ribosome is loaded with the tRNAs non-enzymatically, this approach helps us achieve better cryo-EM density for the A-site tRNA. We added the sentence “Since the tRNAs were loaded non-enzymatically, paromomycin was added to stabilize the A-site tRNA and improve its cryo-EM density.” to the methods section to clarify the reason for adding paromomycin.

3. On page 8, it would be helpful in the text to also mention the amine-carbonyl distance for mABZ. This distance is reported in the discussion and shown in the figure but it may be helpful for the reader to be able to include all three values in the text.

We added these distances into the text on page 10.

4. In the following sentence, "Examples of non-L-α-amino acid monomers that have been introduced into growing polypeptide chains include D-α-amino acids, α-hydroxy and α-thio acids, β2- and β3-amino acids, α-aminoxy and α-hydrazino acids, ...7–15." The authors choose to highlight the work of only one lab (the Suga lab). The authors need to include a broader swath of recent papers and groups in the field, such as: (https://pubs.acs.org/doi/abs/10.1021/jacs.5b12482, https://www.nature.com/articles/s41467-020-18001-x, https://www.nature.com/articles/s41467-022-33701-2, https://pubs.acs.org/doi/10.1021/acscombsci.0c00179, https://pubs.acs.org/doi/10.1021/jacs.8b11521, https://pubs.acs.org/doi/full/10.1021/jacs.5b03135,

https://pubs.acs.org/doi/10.1021/ja035141q)

We are grateful to the reviewer for pointing out some citations that should be added to this sentence. The message we sought to communicate here is that wild type ribosomes can tolerate diverse functionality within the PTC. Some of the papers identified by the reviewer fall in this category. We modified the paragraph to include these works, but in a separate sentence. However, others either require modified ribosomes (Hecht) or enzyme-mediated post-translational reactions (Bowers). These are great papers, but citing them here would go beyond the intent of the sentence.

5. It seems that this paper should be discussed in relation to the current work: https://www.pnas.org/doi/10.1073/pnas.1424712112.

We've added this reference to the Discussion as suggested.

Reviewer: 2

Recommendation: Reconsider after major revisions noted.

Comments:
critical issues:

1. The authors mention the differences in reactivity between Apy, oABZ and mABZ and take this information into account when discussing their models. However, the respective data are not shown. Specifically, no time course of the reaction is shown for any of the derivatives, which is a significant weakness of the paper. The differences in the rate of catalysis between the L-aa and the non-natural derivatives have to be documented by showing the relative time courses of the reaction.

In the prior work from the Suga lab, the authors pre-charged tRNAs with the monomers using flexizymes and monitored the final yields of peptides obtained from IVT reactions, from endpoint assays and mass spectrometry (ref. 13). The present work aims to provide a structural rationale for the differences in overall extent of incorporation the Suga lab observed. We note in the Discussion that other steps of translation could also be affected by the properties of these monomers, including rates of mRNA decoding by the EF-Tu-tRNA ternary complex and tRNA accommodation. We expanded the Discussion to highlight these points that could be pursued in the future. We do not believe that extensive (and time-consuming) time-courses that simply quantify the published trends are needed, especially as we only use the trends to evaluate the cryo-EM structures reported herein.

2. Some of the predictions of the authors' models could be tested by relatively simple biochemical experiments. For example, the suggestion that the disruption of water coordination in the PTC may affect the reaction can be tested using KSIE.

The resolution of the present structures (1.9-2.3 Å) clearly reveals the position of water molecules or lack thereof in the PTC. KSIE experiments are, by contrast, indirect and would not reveal detailed information on solvation in the PTC, as observed in the structures with L-α-amino acid (refs. 5, 16, 28) or with the aminobenzoic acid monomers.

3. For the design of the cryo-EM experiments, it is not entirely clear why there was a need to produce non-hydrolysable tRNA derivatives, as the aminobenzoic acid derivatives are expected to react slowly, which would allow rapid freezing of the cryo-EM sample before any reaction could occur. In Fig. 3A, it is not clear why Met-tRNA was used in the P site, rather than fMet-tRNA as in all other structures, and how this affects the positioning of the substrates in the PTC. In general, the potential effect of the NH replacement (used to avoid premature peptide bond formation) on the tRNA positioning in the PTC should be discussed.

We apologize for the confusion as to why we used amino-tailed tRNAs. Even though the aminobenzoic acid monomers studied here undergo peptidyl transfer within the ribosome less efficiently than a natural L-α-amino acid, they may still be hydrolyzed from the tRNA after charging and prior to accommodation within the ribosome, i.e. during preparation of the cryo-EM samples. This problem pervades the ribosome field, and the amino-tailed tRNAs are used by others, i.e. the Yury Polikanov group, for this reason.

The amino-tailed tRNAs used in this study provide an additional benefit for structural studies. The 3'-amino tail provides a reactive handle for "charging" the tRNAs with the aminobenzoic acid derivatives, molecules that are not known to be charged by natural or mutant amino acid tRNA synthetases. Although flexizyme charging of these monomers has been reported, the acylation yields were found to be low in our hands (ref. 7). The 3'-amino tail on the tRNA allows us to use NHS ester chemistry to install the aminobenzoic acid derivatives onto tRNA in nearly quantitative yields.

In Figure 3A, we compare the position of the non-natural A-site monomer to a previously published high-resolution cryo-EM structure carrying the natural amino acid, Met, in both the P- and A-sites (PDB ID: 8EBB, ref. 20). We use this structure as a standard to compare the position of the reactive groups and PTC nucleotides in the case of ribosome complexes containing only natural amino acids with the position of these moieties in the presently reported structures carrying non-natural monomers in the A-site.

4. The authors refer to the water arrangement in the PTC as "proton wire", however, this is not the only model in the literature, as the authors must be aware of, as they cite a publication supporting and alternative "proton shuttle" model (ref 31). The original "proton wire" model is inconsistent with some biochemical data, whereas "proton shuttle" appears to be compatible with most of the experimental data. I am not sure whether the actual positions of the water molecules are different in the two models, but the mechanism of the proton transfer is different. While the authors of this paper are not supposed to dwell into the alternative models of peptide bond formation for L-amino

acids, it is still important to acknowledge the existence of different models and to take into account the published biochemical results to interpret the structural data.

This point is important, thank you. We broadened the discussion to also include the “proton shuttle” mechanism. The mechanistic arguments remain unchanged.

Reviewer: 3

Recommendation: Publish in ACS Central Science after minor revisions noted.

Comments:

1. Since this paper is under consideration at ACS Central Science, which is meant to target the wide readership of ACS, the authors should consider some more general figure. This could include a zoomed-out version of the part of the ribosome they are referring to when they talk about the distances and the angles between functional groups.

We added a zoomed-out figure showing the large ribosomal subunit that clearly identifies the location of the tRNA positioned in both the P and A sites. We also added a simplified schematic of the peptidyl transferase reaction.

Introduction: If the rationale is to use catalysts that can do multi-turnover reactions (or maybe a greener and natural alternative) to make these polymers, there could be an argument there as to why to start with aminobenzoic derivatives. They explain its uses, but not necessarily why these were useful monomers in their study.

In the Introduction, we now discuss how the backbone of aminobenzoic acids differentiates them – in terms of chemistry, structure, and dynamics – from L-alpha-amino acids. We added the sentence “Yet aminobenzoic acids differ from L-α-amino acids in terms of the conformational flexibility of the backbone atoms (sp^2 vs. sp^3-hybridized) as well as the nucleophilicity of the attacking amine, and the effects of these differences on intra-PTC reactivity are unknown.”

2. p6, L17. In Fig S12E, it looks like the double-headed gray arrow is pointed to the wrong atom in Apy. As per panels F and G, this end of the arrow should be pointed to the carbonyl C of the aromatic ring of the compound, which is to the right not downward. Upon this change, is the distance still 1.6 Å as marked in the manuscript?

We apologize for this error. The figure was updated with the correct distance, which is still 1.6 Å as mentioned in the manuscript.

3. Fig 3B. The dashed line appears to be mispositioned. A per panels C and D, it should go up and to the left so that it connects the nucleophilic amine of Apy and the carbonyl C in the P-site. Upon this change, is the distance still 4.9 Å as marked in the manuscript? This value is used again on p.9 of the ms., so the authors need to be careful. In addition, the BD angles should be provided for B-D. Are they in the 90-110 degree range? This is

hinted at on p8 lines 35-37 but not quantified nor are the (presumably negative) implications for poor BD angles on reactivity discussed.

We reworded the discussion on page 12 to indicate that the carbonyl oxygen is sterically blocked by the position of U2585 in the uninduced state. Since the carbonyl carbon is sterically blocked, the B-D angles become irrelevant. We also fixed the dotted lines in the figure to identify the correct atoms.

4. p8, L31. Shouldn't this be Figure 1C?

Thanks for catching this mistake. We corrected the text to refer to Figure 1D.

5. p9, L1-2. Given the lack of strong structural difference between Apy and oABZ, the authors rightly entertained the notion of different electronic properties. I'd like to suggest two additional considerations.
   (1) Perhaps the endocyclic N in Apy could perform general base catalysis, or if protonated, general acid catalysis and that could promote reactivity. The endocyclic N in the parent pyridine compound is well known to do this. Such activity might help overcome the observed loss of the water wire in the aromatic reagents despite the authors notion that W1 and W2 may not be required for these compounds. The greater dynamics of Apy might allow a docked Apy to do this or alternatively it could do this through buffer catalysis as shown for the twister ribozyme by Messina and Bevilacqua [1].

This idea is interesting and we considered it seriously. We also considered a structural role for the endocytic N, one that results from a discrete H-bond between it (protonated or not) with an atom in the PTC. We see no evidence for such a structural role in any of the three cryo-EM models. Moreover, as the endocyclic N of Apy is located *meta-* to the nucleophilic amine, it points in nearly the opposite direction. This orientation makes it difficult to imagine how it could act as a general acid or base catalyst, even with a more dynamic fMet position.

(2) For Apy and oABZ, there could be an intramolecular hydrogen bond between the exocyclic amine and the carbonyl. Indeed, such compounds often have reduced boiling points because H-bonding is compensated internally (e.g. p-nitrophenol and o-nitrophenol have boiling points of 279 and 216 oC, respectively). This might not explain the difference in reactivity between Apy and oABZ, but it might explain their lower reactivity relative to the natural case.

This is a good point, and we added the sentence "It is also possible that an intramolecular hydrogen bond between the exocyclic amine and carbonyl oxygen further reduces reactivity of these monomers." to the Discussion.

BTW, how much lower they are is not provided but should be.

We now report the relative efficiency of incorporation based on peptide yield determined by the Suga lab in the Introduction (ref. 13).

[1] Messina KJ, Bevilacqua PC. 2018. Cellular small molecules contribute to twister ribozyme catalysis. J Am Chem Soc 140: 10578-10582. doi: 10.1021/jacs.8b06065

6. p2,L24 and again on p10, the authors discuss how in the natural case the induced fit "protects the reaction center from the side chain of the A-site monomer". If this "protection" is lost, one might expect the reactive amine to react elsewhere in the ribosome. Did the authors look for unprotected reactivity of the aminobenzoic acid derivatives with the ribosome?

We reworded these sentences to indicate that before the induced fit is triggered, the ester linkage is shielded from solvent nucleophiles.

oc-2023-00153e.R2

Name: Peer Review Information for "Aminobenzoic acid derivatives obstruct induced fit in the catalytic center of the ribosome"

Second Round of Reviewer Comments

Reviewer: 3

Comments to the Author

The authors did a good job responding to my concerns. Thank you.

Reviewer: 2

Comments to the Author

The authors have answered my question in an acceptable way. While I think that the results of structural studies alone -- regardless at which resolution -- cannot reveal a mechanism and require additional functional studies, the present paper does provide sufficient new important information to publish in ACS Central Science.

Author's Response to Peer Review Comments:

We have formatted the manuscript to address the formatting needs listed above: Reference formatting, TOC graphic, and Synopsis. Please let us know if there any additional issues to address.

Thank you,

Jamie Cate
